# 

**Published:** 1894-07

**Authors:** 


WHOLE NUMBER,

CCCXCIV.

New Series.

July, 1894.

VOL. XXXIII.

No. 12.

### BUFFALO MEDICAL AND SURGICAL JOURNAL.

Established 1845 by AUSTIN FLINT, M. D.

PUBLISHED MONTHLY.

**Editors:**

THOS. LOTHROP, M. D. WM. WARREN POTTER, M. D.

**Associate Editors:**

WILLIAM C. KRAUSS, M. D. JAMES WRIGHT PUTNAM, M. D.
JOHN A. MILLER, PH. D. JOHN PARMENTER, M. D.

SCRIPTA FERUNT ANNOS

EUROPEAN AGENCY,

TRÜBNER & CO.

57 AND 59 LUDGATE HILL, LONDON, E. C.

BUFFALO:
1894.

FOREIGN
SUBSCRIPTION, $2.50

Entered at the Post-Office at Buffalo, N. Y., as Second-class Mail Matter.

*Times Printing House, Buffalo, N. Y.*

Table of Contents on Page V.

